# Prevalence of cervical abnormalities among rural women in KwaZulu-Natal, South Africa

**DOI:** 10.11604/pamj.2022.41.110.27222

**Published:** 2022-02-08

**Authors:** Oluwatosin Motunrayo Omoyeni, Joyce Mahlako Tsoka-Gwegweni

**Affiliations:** 1 Department of Public Health Medicine, School of Nursing and Public Health, College of Health Sciences, University of KwaZulu-Natal, Durban, South Africa,; 2 Office of the Dean, Faculty of Health Sciences, University of the Free State, Bloemfontein, South Africa

**Keywords:** Cervical cancer, cervical abnormalities, cervical screening, Pap smear, intraepithelial lesions, KwaZulu-Natal, South Africa

## Abstract

**Introduction:**

despite the availability of screening facilities in South Africa, cervical cancer prevalence and mortality is still high. Most women present to the health facilities at an advanced stage of disease. This study aimed to determine the prevalence of cervical abnormalities using the revised Bethesda System among rural women in KwaZulu-Natal, South Africa.

**Methods:**

this was a cross-sectional descriptive study using a retrospective medical record review method to collect data on Pap smears from three rural clinics in KwaZulu-Natal. Clinical data and cytology reports were obtained for the years January 2016 to January 2019. Women aged 18-65 years were included.

**Results:**

of 246 randomly-selected medical records, 245 Pap smears were analysed. Half (47.8%) of the women were in the age group 30-44 years. HIV, as a risk factor, was found in 41.2% of the women. A total of 48.6% Pap smears were negative for malignancy. Of the 49.8% abnormal screened results, 25.7% women had low-grade squamous intraepithelial lesion, 13.9% high-grade squamous intraepithelial lesion (HSIL), 8.6% atypical squamous cells of undetermined significance, and 1.6% squamous cell carcinoma (SCC). All SCC cases were found in HIV-infected patients. HSIL and SCC were less common among patients younger than 30 years.

**Conclusion:**

this study´s results accentuate the importance of well-organised cervical screening programmes. Cervical screening, through Pap smears, is a useful, non-invasive and cost-effective method for early detection of pre-invasive lesions. Women, especially those over 30 years, should be educated on the importance of Pap smears and encouraged to uptake the test.

## Introduction

Cervical cancer remains a leading cause of mortality and morbidity among women in South Africa [1, 2]. It is the most common female cancer among black women, with a lifetime risk of 1 in 34 and fourth among white South African women with a lifetime risk of 1 in 93 [3, 4]. It is one of the few preventable cancers where precursor lesions can be identified via screening, and a strong relationship has been established between screening and a decline in mortality of cervical cancer [1]. Similar to most developing countries, the incidence of cervical cancer in South Africa is still inadmissibly high; especially in this modern era where medical science has advanced with well-proven studies on cervical cancer as a preventable disease [2]. Some contributing factors are late diagnosis, high prevalence of HIV/AIDS, poor response to therapy, low ratio of doctors to population, and poor uptake of cervical preventive programmes [1, 5]. Unlike most developing countries, a decrease in mortality and morbidity has been sustained over the years in developed countries - this is as a result of the high quality of screening and coverage. A secondary method of prevention using Pap smears has reduced the incidence of invasive cervical cancer by 70-90% in the developed world [6]. In developing countries, merely 5% of women uptake cervical screening compared to 40-50% in the developed countries [6]. This is evident as 85% of the annual estimated number of women who develop cervical cancer are reported from developing countries [4]. Since five decades ago, opportunistic cervical screening has been offered to South African women. The South African National Department of Health launched the National Guideline for Cervical Screening Programme in 2000. The cervical screening prevention programme offers three free Pap smears in a woman´s lifetime at ten-year intervals, starting from age 30 years for asymptomatic women. The guidelines further specify regular cytology tests for HIV-infected women [1, 5, 7].

Numerous epidemiological studies have proven a strong association between the human papillomavirus (HPV), a sexually transmitted virus, and cervical cancer. Approximately 90% of all cases of cervical cancer are attributed to a certain subtype of HPV [4, 8]. In a study conducted among 22 countries by the International Agency for Research, HPV DNA (99.7%) was identified in practically all the 1,000 cases of cervical cancer [8]. In Southern Africa, the prevalence of HPV infection differs in terms of gender, age group, HIV status, method of collection, and the assay used for examination. The first study on the prevalence of HPV in South Africa was conducted in KwaZulu-Natal between 2004 and 2007. The overall HPV prevalence was 76.3% and 70.5% in the women from rural areas. A similar study on HPV prevalence conducted among women from Gauteng, South Africa, in 2013 showed the prevalence of HPV DNA, high-risk HPV DNA, HPV16 and/or 18 as 74.6%, 54.3% and 19.5%, respectively [2]. A cohort study conducted in 2014 among women from Cape Town (Western Cape Province, South Africa) showed a lesser overall HPV DNA prevalence of 25.4% [2]. In a related study conducted in Sub-Saharan Africa (South Africa, Nigeria and Ghana) between 2007 and 2010, it appeared that the prevalence of single HPV infection, multiple HPV infection and HPV18 is higher in HIV-infected women than non-infected women [2]. Cytological screening with the Pap smear test is an effective and reliable method used for early detection, prevention and delay in cervical cancer progression [9]. Although there are different screening methods in South Africa, cytology-based screening using Pap smear is well established in both private and some public sectors [1]. The Bethesda System has been used for reporting cervical cytology (Pap smear) results for about 30 years [10, 11]. Its standardised framework and terminology has promoted global unification of clinical management of cervical lesions among clinicians and laboratorians [10, 11]. In South Africa, few studies have been conducted using the revised Bethesda System to determine the prevalence of cervical cancer among rural women in this country. Therefore, this study intended to determine the prevalence of cervical abnormalities using the revised Bethesda System among rural women in South Africa. The objectives were to (i) provide a socio-demographic profile of rural women who present for a Pap smear at rural clinics in South Africa; (ii) compare demographics with Pap smear results; and (iii) compare risk factors and clinical symptoms with Pap smear results.

## Methods

**Study design and setting:** this was a cross-sectional descriptive study that used a retrospective medical record review method to collect data from three primary healthcare clinics located at the Valley of Thousand Hills, a rural settlement in KwaZulu-Natal, South Africa. The residents of most villages in this settlement are poor and unemployed. The development of the community is restricted by factors such as poor health services, large families and inadequate infrastructure. KwaZulu-Natal is one of the provinces with the highest incidence of HIV in South Africa, placing additional pressures on people in the area [12]. The study evaluated 246 Pap smear results of all women screened between 1^st^ January 2016 and 31^s^^t^ January 2019.

**Study population and sampling:** medical records of all women aged 18 to 65 years who underwent Pap smears at any of the three selected rural clinics between 1^st^ January 2016 and 31^st^ January 2019 were included in the study.

**Sample size:** the sample size for the medical record review was arrived at using the sample size calculation method, with 80% power (1-β [type 2 error probability]) and 95% confidence (or a 5% error probability [type 1]), assuming a baseline prevalence proportion of 20%. To account for 15% non-response, a total of 246 medical records were reviewed.

**Data collection:** of the 1 032 potentially eligible patients, the medical records of 246 patients were randomly selected from the three rural clinics using a computer-generated table from the cervical cancer screening register. Medical records were reviewed to obtain relevant data on the prevalence of cervical cancer using the folder number of patients, who have been screened, to trace their case file. Relevant data extracted from the case files were age, marital status, parity, level of education, occupation, predisposing factors to cervical cancer, previous screening methods used, clinical features of cervical cancer and screening results. Data from the medical records were collected at the three rural clinics between 21^st^ November 2018 and 31^st^ January 2019. The laboratory used the revised Bethesda System of classification for reporting Pap smear results. The system generally classifies the smear as “satisfactory for evaluation” or “unsatisfactory for evaluation”. The system further categorises lesions broadly as “negative either for intraepithelial lesions or malignancy” or “epithelial cell abnormalities”.

**Data analysis:** data were collected by the principal investigator and entered into a Microsoft Excel spreadsheet. Data were processed and analysed using SPSS V26. Categorical explanatory variables were cross-tabulated against dichotomous outcomes. Categorical data are presented using frequency distribution tables.

**Ethical consideration:** ethics approval was obtained from Biomedical Research Ethics Committee (BREC Ref No: BE227/18) and permission to conduct this study was obtained from the KwaZulu-Natal Department of Health. Participant´s informed consent was not required as this was a record review.

## Results

A total of 246 medical records were reviewed; one medical record was excluded in the study analysis due to incomplete information, therefore 245 medical records were included in the analysis.

### Demographic characteristics of patients

The demographic characteristics of the women are presented in Table 1. Out of 245 women, approximately half (47.8%) were aged 30 to 44 years. The majority of the women were single (72.5%). Most (76.8%) had attended secondary school, while 9.5% did not have any formal education. Only 3.7% of the women had no children, while 61.7% had between two and four children. Most of the women were unskilled workers (65.4%). There were no skilled workers.

**Table 1:** demographic characteristics of study participants

Variables and categories	Frequency	Percentage
	N	%
**Age group (years) (n = 245)**		
< 30	79	32.2
30-44	117	47.8
≥ 45	49	20.0
**Marital status (n = 244)**		
Single	177	72.5
Married	67	27.5
**Educational status (n = 241)**		
No formal education	23	9.5
Primary education	25	10.4
Secondary education	185	76.8
Tertiary education	8	3.3
**Parity (n = 243)**		
0	9	3.7
1	67	27.6
2-4	150	61.7
5+	17	7.0
**Occupation (n = 234)**		
Unemployed	76	32.5
Unskilled	153	65.4
Semi-skilled	5	2.1

### Demographic characteristics and results of pap smear

The revised Bethesda System was used to present the results of the Pap smears. Half (48.6%) of Pap smears were negative for malignancy (NILM). Of the abnormal screened results (49.8%), low-grade squamous intraepithelial lesion (LSIL) was the commonest abnormality identified (25.7%), followed by high-grade squamous intraepithelial lesion (HSIL) (13.9%), 8.6% had atypical squamous cells of undetermined significance (ASC-US) and 1.6% had squamous cell carcinoma (SCC). The remaining 1.6% was unspecified and represents the results marked as “diagnosis deferred”, i.e. the laboratory picture of the smear could not be explained. Table 2 summarises the relationship between socio-demographic characteristics and results of the Pap smears. About half of the women in each age group had NILM. Women aged ≥ 45 years had the highest percentage of ASC-US at 16.3%. The likelihood of having a positive smear for LSIL decreased with age, < 30 (36.7%), aged 30-44 (22.2%) and ≥ 45 years (16.3%). The age group 30-44 years had the highest percentage of HSIL at 19.7%. The age group ≥ 45 years seems to be the most affected (4.1%) by SCC. A higher percentage of married women (62.7%) had NILM compared to single women (43.5%). Married women had a slightly higher percentage of ASC-US (9.0%) and SCC (3.0%). Single women had the highest percentage of LSIL (29.4%) and HSIL (15.8%).

**Table 2:** demographic characteristics and results of the Pap smears

Variables and categories	NILM	ASC-US	LSIL	HSIL	SCC	Unspecified	n (%)
**Age group (years) (n = 245)**							
< 30	46.8	8.9	36.7	6.3	0.0	1.3	79 (32.2)
30-44	50.4	5.1	22.2	19.7	1.7	0.9	117 (47.8)
≥ 45	46.9	16.3	16.3	12.2	4.1	4.1	49 (20.0)
**Marital status (n = 244)**							
Single	43.5	8.5	29.4	15.8	1.1	1.8	177 (72.5)
Married	62.7	9.0	16.4	7.5	3.0	1.5	67 (27.5)
**Educational status (n = 241)**							
No formal education	43.5	13.0	21.7	8.7	0.0	13.0	23 (9.5)
Primary education	44.0	16.0	20.0	20.0	0.0	0.0	25 (10.4)
Secondary education	50.8	7.6	25.4	13.5	2.2	0.5	185 (76.8)
Tertiary education	12.5	0.0	75.0	12.5	0.0	0.0	8 (3.3)
**Parity (n = 243)**							
0	55.6	0.0	33.3	11.1	0.0	0.0	9 (3.7)
1	47.8	10.4	31.3	7.5	1.5	1.5	67 (27.6)
2-4	47.3	8.0	24.0	16.7	2.0	2.0	150 (61.7)
5+	64.7	11.8	11.8	11.8	0.0	0.0	17 (7.0)
**Occupation (n = 234)**							
Unemployed	44.7	9.2	35.5	6.6	1.3	2.6	76 (32.5)
Unskilled	51.0	7.2	22.2	17.0	2.0	0.7	153 (65.4)
Semi-skilled	20.0	0.0	40.0	40.0	0.0	0.0	5 (2.1)

ASC-US = Atypical squamous cells of undetermined significance; HSIL = High-grade squamous intraepithelial lesion; LSIL = Low-grade squamous intraepithelial lesion; NILM = Negative for intraepithelial lesion or malignancy; SCC = Squamous cell carcinoma

About half of women with no formal education (43.5%), primary education (44.0%) and secondary education (50.8%) had NILM. Women with tertiary education recorded the highest percentage of those diagnosed with LSIL (75.0%); those with secondary education and with no formal education accounted for 25.4% and 21.7%, respectively. Women with primary education had the highest percentage of ASC-US at 16.0% and HSIL at 20.0%. Women with secondary education seem to be the most affected by SCC at 2.2%. Regardless of parity, the percentage of women whose Pap smear test revealed NILM was ≥ 50%. Women with 5+ children had the highest percentage of ASC-US (11.8%). The screening results revealed that women with parity zero had the highest number of LSIL at 33.3%. Similarly, women with more than one birth tended to have a higher percentage of HSIL than those who had one child or none, parity 2-4 (16.7%), parity 5+ (11.8%) vs parity 1 (7.5%) and parity zero (11.1%). Half (51.0%) of unskilled women had NILM, the shares of the unemployed and semi-skilled women were 44.7% and 20.0%, respectively. Unemployed women had the highest percentage of ASC-US at 9.2% while the semi-skilled women had the highest percentage of LSIL (40.0%) and HSIL (40.0%). Unskilled women had the highest percentage of SCC (2.0%).

### Risk factors/clinical symptoms and results of pap smear

Table 3 summarises the relationship between risk factors/clinical symptoms and results of the Pap smears. While 46.9% of the women had no identifiable risk factors, the remaining 53.1% had risk factors that included HIV-positive (41.2%), a sexually transmitted infection (STI) (4.1%), early sexual debut (3.3%), a family history of cervical cancer (2.9%) and two or more children (1.6%). The results indicated that a high proportion of women (75.0%) who reported early sexual debut had NILM; this is in comparison with 50.0% diagnosed with STI, 50.0% with multiple births, 44.6% with HIV and 28.6% with a family history of cervical cancer. Women with early sexual debut had the highest percentage of ASC-US at 12.5%. The risk factor with the highest percentage of women with LSIL was HIV (26.7%). A family history of cervical cancer appeared to be an important risk factor, with an estimated 57.1% for HSIL. As per the clinical symptoms, 74.0% of the women had no identifiable clinical symptoms. Vaginal discharge was the commonest clinical symptom (12.2%), followed by bleeding per vagina (7.8%) and low abdominal pain (6.1%). Low abdominal pain had the highest percentage of ASC-US (13.3%). Patients with bleeding had the highest percentage of LSIL (31.6%) and HSIL (26.3%). No SCC was seen for vaginal discharge, bleeding per vagina and low abdominal pain. On the 76 (31.0%) comments from Pap smear results, the endocervical component was absent or insufficient in 15.9% of Pap smears, 7.3% had bacterial vaginosis, 4.1% had trichomonas vaginalis, 2.0% had candida species and 1.6% of the smears were not satisfactory for evaluation.

**Table 3:** risk factors, clinical symptoms and results of the Pap smears (N = 245)

Variables and categories	NILM	ASC-US	LSIL	HSIL	SCC	Unspecified	n (%)
**Risk factor**							
HIV	44.6	10.9	26.7	11.9	4.0	2.0	101 (41.2)
STI	50.0	10.0	20.0	20.0	0.0	0.0	10 (4.1)
Family history	28.6	0 .0	14.3	57.1	0.0	0.0	7 (2.9)
Early sexual debut	75.0	12.5	12.5	0.0	0.0	0.0	8 (3.3)
Multiparity (≥ 2 children)	50.0	0.0	0.0	50.0	0.0	0.0	4 (1.6)
No identifiable risk factor	51.3	7.0	27.8	12.2	0.0	1.7	115 (46.9)
**Clinical feature**							
Vaginal discharge	50.0	10.0	26.7	13.3	0.0	0.0	30 (12.2)
Bleeding per vagina	31.6	10.5	31.6	26.3	0.0	0.0	19 (7.8)
Low abdominal pain	60.0	13.3	6.7	20.0	0.0	0.0	15 (6.1)
No identifiable clinical symptom	49.2	7.7	26.5	12.2	2.2	2.2	181 (73.9)
**Comment from Pap smear result**							
Endocervical component absent/Insufficient	71.8	7.7	17.9	0.0	2.6	0.0	39 (15.9)
Bacterial vaginosis	66.7	16.7	16.7	0.0	0.0	0.0	18 (7.3)
Trichomonas vaginalis	50.0	10.0	30.0	10.0	0.0	0.0	10 (4.1)
Candida species	80.0	0.0	20.0	0.0	0.0	0.0	5 (2.0)
Not satisfactory for evaluation	0.0	0.0	25.0	0.0	0.0	75.0	4 (1.6)
No comment	41.4	8.3	28.4	19.5	1.8	0.6	169 (69.0)

ASC-US = Atypical squamous cells of undetermined significance; HSIL = High-grade squamous intraepithelial lesion; LSIL = Low-grade squamous intraepithelial lesion; NILM = Negative for intraepithelial lesion or malignancy; SCC = Squamous cell carcinoma; STI - Sexually transmitted infection

## Discussion

Over the last three decades, the incidence of cervical cancer has reduced due to widespread cytology-based cervical cancer screening using Pap smears. In view of the proven efficacy of Pap smears in the detection of pre-invasive cervical lesions, cervical screening is advocated to commence in all asymptomatic South African women starting at 30 years of age [13]. The results of the present study showed that almost half (47.8%) of the screenings were done among women aged 30 to 44 years. This finding is comparable to similar studies conducted in Nepal, Mid-Western Nepal, India and Nigeria, with the highest percentage screening done among patients in the age group 30 to 39 (39.0%), 30 to 39 (40.23%), 31 to 40 (32.68%) and age 40 years and above (65.9%) [9, 13-15]. HIV represented a major risk factor in our study (41.2%). In a related retrospective, hospital-based, study conducted in Nepal on Pap smear and its clinical correlation, 456 medical records were reviewed. Early marriage with early exposure to sexual activity (32.0%) was recorded as the highest risk factor, followed by multiple sexual partners (4.6%) [9]. In a similar study conducted in KwaZulu-Natal, South Africa, HIV prevalence among study participants was 21% [16]. Also, a comparable study from Nigeria showed an HIV prevalence of 4.8% with a prevalence of abnormal Pap smear results of 25.0% among HIV-positive women compared to 22.5% among HIV-negative women [15].

Sexually transmitted agents, such as HIV, herpes simplex virus and chlamydia trachomatis are some cofactors expected to affect the progression of cervical lesions to high-grade lesions and invasive cervical cancer [17]. HIV has been associated with a high risk of cervical cancer in many studies. Women who are HIV positive have been reported to present 10 years earlier with invasive cervical cancer than women who are HIV negative. Furthermore, patients who are HIV infected are more likely to present with late stage of the disease at first diagnosis than non-HIV infected patients. The risk of cervical cancer has been shown to be 10 times more in severely immunocompromised patients (with a CD4 cell count less than 200/μL) than those with a CD4 cell count of 200/μL and above [18]. Vaginal discharge was the commonest clinical symptom in our study (12.2%), followed by bleeding per vagina (7.8%) and low abdominal pain (6.1%). Similar findings were reported in a study with vaginal discharge as the major presentation 34.6%, post-coital bleeding at 11.4% and abdominal pain at 16.9% [9]. Other similar studies also found vaginal discharge as the commonest symptom [14, 19, 20]. The leading cause of atypical vaginal discharge among reproductive-age women is bacterial vaginosis. In this study, 7.3% of patients were diagnosed with bacterial vaginosis. Unlike vaginal candidiasis, (2.0% from this study), there is a positive association between bacterial vaginosis and an increased risk of cervical pre-cancerous lesions [21]. However, there have been debates and inconsistent evidence as regards to the association between cervical pre-cancerous lesions and bacterial vaginosis. Bacterial vaginosis has, however, been proposed to play a role in the development of cervical cancer [9, 17, 21].

Of the 245 pap smears reviewed, 49.8% smears were reported positive for cervical epithelial abnormalities. Low-grade squamous intraepithelial lesion is the commonest identified abnormality in our study at 25.7%, followed by HSIL (13.9%), with SCC the least diagnosed at 1.6%. Similar patterns were found in a study conducted in India, where LSIL was recorded as the most frequent diagnosed epithelia abnormality, followed by HSIL with SCC the least diagnosed epithelia abnormality [10]. Contrary to our study, ASC-US was found to be the most common epithelial cell abnormality in several published studies [9, 14, 22, 23], whereas a low 8.6% of women had ASC-US. Atypical squamous cells have been shown to be by far the category of abnormal cervical cytology interpretation commonly reported [11]. In agreement with the current study, SCC or invasive carcinoma is reported with low numbers from similar studies from other countries [9, 14, 22, 23]. This low number of SCC could be explained by well-established screening programmes, which have been shown to promote early detection and treatment of abnormal precursor lesions [22]. Low-grade squamous intraepithelial lesion was most prevalent (36.7%) among women under 30 years of age in our study; this is comparable to a similar study conducted among women attending clinics in Nigeria, where LSIL was found to predominate in the age group 20 to 29 years [15]. In another study conducted in Brazil, women in the age group 15 to 30 years were the most diagnosed with LSIL [23].

High-grade squamous intraepithelial lesion seems to predominate among women aged 30 to 44 years in the current study, which is close to the usual age distribution of HSIL of around 35 to 45 years [6]. In similar studies conducted in South Africa and Nigeria, women diagnosed with HSIL were younger than 30 years and aged 40 and above, respectively [6, 15]. The oldest age group in this study (age 45 and above) was the percentage of women most diagnosed with SCC (1.6%). Squamous cell carcinoma predominated in patients aged 40 years and above in similar studies conducted in Nigeria and Saudi Arabia [15, 23]. All cases of SCC in this study occurred among HIV-infected patients, which further suggests a strong association between cytological changes and HIV infection [6].

Women with secondary education and primary education were most diagnosed with HSIL and SCC, respectively, in the current study. Educational level is a variable that has been found to be associated with the uptake of Pap smear, although not statistically significant in all studies [24]. A high screening rate has been found in women with a high level of education in several studies, however, a few studies have shown that women with a high level of education may not necessarily go for screening [24, 25]. Even though education is an essential tool in health promotion with regard to cervical screening, it is not a solitary factor influencing health practices of women [26]. Women with more than one birth had a higher percentage of HSIL than those who had one child or none in this study. Multiparity has been associated with a greater risk of invasive cervical cancer compared to women with no offspring [27]. It was observed in the current study and similar studies that women with children are more likely to take up cervical screening than those with no children. This could be explained by the interaction between women who attend maternal and child health clinics and their health care practitioner [24, 27]. In addition, the women who attend these clinics may be more receptive to reproductive health care due to gained information and experience [24, 27].

**Study limitations:** the study is limited by the fact that data were collected from one area of rural KwaZulu-Natal, therefore, the study may be restricted as regards to generalisability. However, facilities in rural South Africa are similar, thus the study may be applicable to other rural settings. The study being retrospective, it is difficult to ascertain the eventual outcome of patients. In addition, patients were referred to tertiary institutions for further management, hence the pattern of disease could not be established.

## Conclusion

This study described the prevalence of cervical abnormalities in rural South Africa. The most common abnormalities were LSIL, HSIL and ASC-US. The main risk factors were HIV and STI. Vaginal discharge and bleeding per vagina were the most common clinical findings in this study. Women of an older age group seemed to be most affected with HSIL and SCC. Cervical screening (Pap smear) is a useful, non-invasive and cost-effective screening method for early detection of pre-invasive lesions. Women, especially those above 30 years, should be educated on the importance of a Pap smear and encouraged to uptake the test. The significance of routine screening for HIV-infected women cannot be overemphasised, as HIV represents a serious risk factor. Similarly, women who present with unusual vaginal discharge and/or unexplained bleeding per vagina should be encouraged to undergo cervical screening, as these are prime symptoms of cervical abnormalities.

### What is known about this topic

- Cervical cancer has contributed considerably to high morbidity and mortality in poor-resource settings;
- The incidence of cervical cancer has declined in the past three decades as a result of cervical cytology screening (Pap smear).

### What this study adds

- The study supports the current cervical cancer screening guidelines in South Africa to screen older women as they are the most affected with HSIL and SCC;
- Irrespective of age, women with risk factors (HIV, recurrent STI, family history of cervical cancer, etc.) and clinical symptoms (unusual vaginal discharge, bleeding per vaginal, recurrent lower abdominal pain, etc.) are predisposed to developing cervical cancer and should be encouraged to screen for cervical cancer.

## References

[1] Botha MH, Dreyer G (2017). Guidelines for cervical cancer screening in South Africa. South A J Gynaecol Oncol.

[2] Jordaan S, Michelow P, Richter K, Simoens C, Bogers J-P (2016). A review of cervical cancer in South Africa: previous, current and future. Health Care Current Reviews.

[3] Moodley M (2009). Cervical cancer in Southern Africa: The challenges. South A J Gynaecol Oncol.

[4] Hailemariam T, Yohannes B, Aschenaki H, Mamaye E, Orkaido G, Seta M (2017). Prevalence of cervical cancer and associated risk factors among women attending cervical cancer screening and diagnosis center at Yirgalem General Hospital, Southern Ethiopia. J Cancer Sci Ther.

[5] Botha MH, Richter KL (2015). Cervical cancer prevention in South Africa: HPV vaccination and screening both essential to achieve and maintain a reduction in incidence. S A Med J.

[6] Gaym A, Mashego M, Kharsany AB, Walldorf J, Frohlich J, Karim QA (2007). High prevalence of abnormal Pap smears among young women co-infected with HIV in rural South Africa-implications for cervical cancer screening policies in high HIV prevalence populations. S A Med J.

[7] South African National Department of Health (2012). National guideline for cervical screening programme.

[8] Milutin-Gašperov N, Sabol I, Halec G, Matovina M, Grce M (2007). Retrospective study of the prevalence of high-risk human papillomaviruses among Croatian women. Coll Antropol.

[9] Sharma GD, Adhikari R, Parajuly SS, Adhikari KG (2019). Cervical cancer screening in a tertiary care centre by Pap smear and its clinical correlation. Medical Journal of Pokhara Academy of Health Sciences.

[10] Nair GG, Shamsuddin F, Narayanan T, Balan P (2016). Cytopathological pattern of cervical pap smears-a study among population of North Malabar in Kerala. Indian J Pathol Oncol.

[11] Nayar R, Wilbur DC (2015). The Pap Test and Bethesda 2014. The reports of my demise have been greatly exaggerated (after a quotation from Mark Twain). Acta Cytol.

[12] Rossiter G, Twala N, Sebastian B (2007). Prepared by Khulisa Management Services. A Case Study. The Valley Trust OVC Project.

[13] Ranabhat S, Shrestha R, Tiwari M (2011). Analysis of abnormal epithelial lesions in cervical Pap smears in Mid-Western Nepal. Journal of Pathology of Nepal.

[14] Bamanikar SA, Baravkar DS, Chandanwale SS, Dapkekar P (2014). Study of cervical Pap smears in a tertiary hospital. Indian Medical Gazette.

[15] Duru CB, Oluoha UR, Uwakwe K, Diwe KC, Merenu IA, Emerole CA (2015). Pattern of Pap smear test results among Nigerian women attending clinics in a teaching hospital. Int J Curr Microbiol App Sci.

[16] Moodley M, Mould S (2005). Invasive cervical cancer and human immunodeficiency virus (HIV) infection in KwaZulu-Natal, South Africa. J Obstet Gynaecol.

[17] Gillet E, Meys JF, Verstraelen H, Verhelst R, De Sutter P, Temmerman M (2012). Association between bacterial vaginosis and cervical intraepithelial neoplasia: systematic review and meta-analysis. PloS One.

[18] Heard I, Tassie J-M, Schmitz V, Mandelbrot L, Kazatchkine MD, Orth G (2000). Increased risk of cervical disease among human immunodeficiency virus-infected women with severe immunosuppression and high human papillomavirus load. Obstet Gynecol.

[19] Pradhan B, Pradhan SB, Mital VP (2007). Correlation of Pap smear findings with clinical findings and cervical biopsy. Kathmandu Univ Med J (KUMJ).

[20] Solomon D, Nayar R (2004). The Bethesda system for reporting cervical cytology. Definitions, criteria, and explanatory notes.

[21] Rodriguez-Cerdeira C, Sanchez-Blanco E, Alba A (2012). Evaluation of association between vaginal infections and high-risk human papillomavirus types in female sex workers in Spain. ISRN Obstet Gynecol.

[22] Altaf FJ, Mufti ST (2012). Pattern of cervical smear abnormalities using the revised Bethesda system in a tertiary care hospital in Western Saudi Arabia. Saudi Med J.

[23] Abdullah LS (2007). Pattern of abnormal Pap smears in developing countries: A report from a large referral hospital in Saudi Arabia using the revised 2001 Bethesda System. Ann Saudi Med.

[24] Gan DE, Dahlui M (2013). Cervical screening uptake and its predictors among rural women in Malaysia. Singapore Med J.

[25] Lyimo FS, Beran TN (2012). Demographic, knowledge, attitudinal, and accessibility factors associated with uptake of cervical cancer screening among women in a rural district of Tanzania: three public policy implications. BMC Public Health.

[26] Okunowo AA, Daramola ES, Soibi-Harry AP, Ezenwankwo FC, Kuku JO, Okunade KS (2018). Women's knowledge of cervical cancer and uptake of Pap smear testing and the factors influencing it in a Nigerian tertiary hospital. Journal of cancer Research and Practice.

[27] Ncube B, Bey A, Knight J, Bessler P, Jolly PE (2015). Factors associated with the uptake of cervical cancer screening among women in Portland, Jamaica. N Am J Med Sci.

